# Temporal voice areas exist in autism spectrum disorder but are dysfunctional for voice identity recognition

**DOI:** 10.1093/scan/nsw089

**Published:** 2016-06-30

**Authors:** Stefanie Schelinski, Kamila Borowiak, Katharina von Kriegstein

**Affiliations:** ^1^Max Planck Institute for Human Cognitive and Brain Sciences, Max Planck Research Group, Neural mechanisms of human communication, Leipzig, 04103, Germany; ^2^Berlin School of Mind and Brain, Humboldt University of Berlin, Berlin, 10117; ^3^Department of Psychology, Humboldt University of Berlin, Berlin, 12489, Germany

**Keywords:** autism spectrum disorder, voice recognition, auditory, person identity recognition, superior temporal sulcus

## Abstract

The ability to recognise the identity of others is a key requirement for successful communication. Brain regions that respond selectively to voices exist in humans from early infancy on. Currently, it is unclear whether dysfunction of these voice-sensitive regions can explain voice identity recognition impairments. Here, we used two independent functional magnetic resonance imaging studies to investigate voice processing in a population that has been reported to have no voice-sensitive regions: autism spectrum disorder (ASD). Our results refute the earlier report that individuals with ASD have no responses in voice-sensitive regions: Passive listening to vocal, compared to non-vocal, sounds elicited typical responses in voice-sensitive regions in the high-functioning ASD group and controls. In contrast, the ASD group had a dysfunction in voice-sensitive regions during voice identity but not speech recognition in the right posterior superior temporal sulcus/gyrus (STS/STG)—a region implicated in processing complex spectrotemporal voice features and unfamiliar voices. The right anterior STS/STG correlated with voice identity recognition performance in controls but not in the ASD group. The findings suggest that right STS/STG dysfunction is critical for explaining voice recognition impairments in high-functioning ASD and show that ASD is not characterised by a general lack of voice-sensitive responses.

## Introduction

The ability to recognise the identity of another person develops very early in infancy (De Casper and Fifer, 1980; Kisilevsky *et al.*, 2003) and is a key requirement for human interaction (Bruce and Young, 1986; Ellis *et al.*, 1997; Belin *et al.*, 2004; von Kriegstein *et al.*, 2008; Blank *et al.*, 2014). Also, many other species recognise the identity of conspecifics (Tibbetts and Dale, 2007; Proops *et al.*, 2009; Sliwa *et al.*, 2011)—an ability often critical for survival (Jouventin *et al.*, 1999; Searby and Jouventin, 2003; Levey *et al.*, 2009; Magrath *et al.*, 2009; McComb *et al.*, 2014).

A large proportion of the human population suffers from person recognition impairments, including difficulties in recognising faces or voices (Kennerknecht *et al.*, 2006; Kennerknecht *et al.*, 2008; Roswandowitz *et al.*, 2014). Autism spectrum disorder (ASD), which is characterised by difficulties in communication and social interaction (DSM-5, American Psychiatric Association, 2013), is associated with both, impaired face (for review see Weigelt *et al.*, 2012) and voice recognition (Boucher *et al.*, 1998; Schelinski *et al.*, 2014; Schelinski *et al.*, in press). A large number of neuroimaging studies have investigated the face processing and face recognition impairment in ASD and revealed an altered functioning of visual association cortices specialised for faces (Schultz *et al.*, 2000; Pierce *et al.*, 2001; Pierce *et al.*, 2004; Kleinhans *et al.*, 2008; Domes *et al.*, 2013). Much less is known about the voice processing and recognition difficulties in ASD (Gervais *et al.*, 2004).

The investigation of the neuronal profile of voice recognition in ASD is important for two reasons. First, person perception deficits are socially restricting (Yardley *et al.*, 2008; Fine, 2012) and difficulties in recognising other persons likely add to the communication difficulties that are a core feature of ASD. Characterising voice recognition difficulties will increase our knowledge about the challenges people with ASD are faced with in social situations. Second, knowledge of the neuronal mechanisms will not only enhance our understanding of the functional neuropathology of ASD but also inform neuroscientific models of person recognition (Ellis *et al.*, 1997; Belin *et al.*, 2004; Young and Bruce, 2011; Blank *et al.*, 2014).

Here, we used functional magnetic resonance imaging (fMRI) to systematically investigate the neural mechanisms of voice processing and their relation to behavioural performance in a group of adults with high-functioning ASD and typically developed matched controls. In the first fMRI experiment, we used a standard fMRI protocol for investigating voice processing (Belin *et al.*, 2000). In this experiment, participants passively listened to blocks of vocal (e.g. coughing, speech) and non-vocal sounds (e.g. car or bell sounds) (Belin *et al.*, 2000). Typically developed individuals usually show higher responses to vocal than to non-vocal sounds in the so-called temporal voice areas (TVA), voice-sensitive areas that are located in the bilateral superior temporal sulcus and gyrus (STS/STG; Belin *et al.*, 2000; Belin *et al.*, 2002; von Kriegstein and Giraud, 2006). A pioneering fMRI study (Gervais *et al.*, 2004) showed that four of the five tested participants with ASD had no significant blood oxygenation level-dependent (BOLD) response in the TVA, as compared to non-vocal, sounds (*P* < 0.001 uncorrected). Such general lack of responses for vocal sounds is surprising because it contrasts the behavioural pattern found in ASD that indicates a selective deficit for the recognition of unfamiliar voices: people with high-functioning ASD have difficulties in recognising unfamiliar but not familiar voices (Schelinski *et al.*, in press). In addition, the impaired voice identity recognition is dissociable from intact speech recognition (Schelinski *et al.*, 2014). Our first aim was, therefore, to replicate the findings by Gervais *et al.* (2004) in a larger and well-matched ASD and control group. Our second aim was to investigate the neural mechanisms of voice identity recognition in ASD. To do this, we conducted a second fMRI experiment, in which participants learned novel voices and performed voice identity and speech recognition tasks on the same stimulus material (von Kriegstein *et al.*, 2003; von Kriegstein and Giraud, 2004).

## Materials and methods

### Participants

We tested 16 adults with ASD (ASD group) and 16 typically developed individuals (control group). The groups were matched pairwise with respect to gender, chronological age, handedness (Oldfield, 1971) and intelligence quotient (IQ) (Table 1). IQ was assessed using the German adapted version of the Wechsler Adult Intelligence Scale (Wechsler, 1997; German version by von Aster *et al.*, 2006). All participants had an IQ within the normal range or above (defined as a full-scale IQ of at least 85), indicating that all participants were on a ‘high-functioning’ cognitive level. A full-scale IQ difference within each subject pair was maximally 1 s.d. (15 IQ points). Additionally, groups showed comparable concentration performances (d2 test of attention; Brickenkamp, 2002; Table 1).

**Table 1.** Descriptive statistics for the ASD (n = 16) and the control group (n = 16) and group comparisons. Each participant in the control group was matched with respect to chronological age, gender, intelligence quotient (IQ), and handedness to the profile of one ASD participant

	ASD		Controls		
Gender	13 males, 3 females		13 males, 3 females		
Handedness^a^	14 right, 2 left		14 right, 2 left		
	*M*	SD	*M*	*SD*	*P*
Age	33.75	10.12	33.69	9.58	0.986
Range	20–51		18–52		
WAIS^b^ scales					
Full-scale IQ	110.31	13.79	111.50	10.97	0.789
Verbal IQ	110.75	12.35	108.75	12.59	0.653
Performance IQ	107.38	17.55	112.69	9.59	0.296
Working memory	108.63	2.22	108.00	3.76	0.887
Concentration^c^	104.19	8.61	106.06	3.41	0.645
AQ^d^	39.81	6.61	14.13	4.77	<0.001*
Range	26–48		5–23		

^a^Handedness was assessed using the Edinburgh handedness questionnaire (Oldfield, 1971).

^b^WAIS = Wechsler Adult Intelligence Scale (Wechsler, 1997; German adapted version: von Aster *et al.*, 2006; *M* = 100; SD = 10).

^c^Concentration = d2 Test of Attention (Brickenkamp, 2002; *M* = 100; SD = 10).

^d^AQ = Autism Spectrum Quotient (Baron-Cohen *et al.*, 2001).

*Significant group difference (*P* < 0.05). M = mean; SD = standard deviation.

All participants reported normal hearing abilities and no limitations or disorders associated with the ear or hearing. Normal hearing abilities were confirmed with pure tone audiometry (hearing level equal or above 25 dB at the frequencies of 250, 500, 1000, 1500, 2000, 3000, 4000, 6000 and 8000 Hz). All participants were native German speakers. All were free of medication except two participants taking histamine antagonist against allergies (one control and one ASD) and two participants taking blood pressure medication (two ASD). None of the participants reported to have a neurological disease. Three additional individuals in the ASD group were not included in the analysis due to incidental findings in an anatomical MRI scan. We also excluded the control participants who were matched to these ASD participants’ profiles.

We recruited individuals in the ASD group via autism outpatient clinics and announcements in ASD communities. Participants in the ASD group had previously received a formal clinical diagnosis of Asperger syndrome (12 males and 2 females) or childhood autism (2 males, Verbal-IQ 100 and 119) according to the diagnostic criteria of the International and Statistical Classification of Diseases and Related Health Problems (ICD-10; World Health Organization, 2004). Additionally, the diagnoses for all ASD group participants were confirmed with the Autism Diagnostic Observation Schedule (Lord *et al.*, 2000; German version by Rühl *et al.*, 2004) and, if caregivers were available (*n* = 9), using the Autism Diagnostic Interview-Revised (ADI-R; Lord *et al.*, 1994; German version by Bölte *et al.*, 2003) and Social Communication Questionnaire (SCQ; Rutter *et al.*, 2003; German version by Bölte and Poustka, 2006) (Supplementary Table S1).

We recruited the control group participants from the participant database of the Max Planck Institute for Human Cognitive and Brain Sciences Leipzig. Participants in the control group reported to have no neurological or psychiatric history and no family history of ASD. None of the controls exhibited a clinically relevant number of traits associated with ASD as assessed by the Autism Spectrum Quotient (Baron-Cohen *et al.*, 2001; Table 1).

All participants received payment for their participation. The study was approved by the Ethics Committee of the Medical Faculty at the University Leipzig, Germany (299-12-14092012). All participants gave written informed consent.

### Experiments

**Fig. 1.**
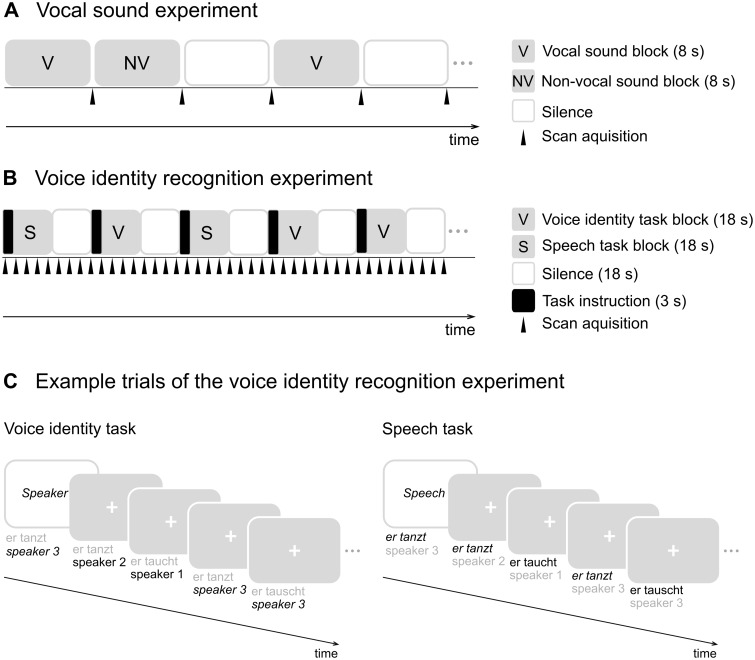
Experimental design of the two fMRI experiments. (A) In the vocal sound experiment, participants listened to blocks of vocal sounds (V), non-vocal sounds (NV), and silence (white boxes). One brain volume was acquired after each block. (B) In the voice identity recognition experiment, there were two conditions. In one condition, participants had to recognise who was speaking (voice identity task). In the other condition, participants had to recognise what was said (speech task). Stimuli consisted of blocks of 13 auditory sentences. At the beginning of each block, a key-word (‘Speaker’, ‘Speech’) on the screen instructed the participants to perform the voice identity or the speech task. Scans were acquired continuously. (C) Example trials of the voice identity recognition experiment: Participants decided for each sentence whether it was spoken by the target speaker (voice identity task) or whether it matched the content of the target sentence (speech task). Stimuli in the voice identity and speech task blocks were the same.

Our study included two fMRI experiments (Figure 1): (i) vocal sound experiment and (ii) voice identity recognition experiment. For participants who never had an MRI scan before, we conducted a mock MRI scan to familiarise participants with the MRI environment. This was done on a separate day before the actual MRI scanning. Stimuli during the fMRI sessions from both experiments were presented using a MR confon system (Mark II; MR confon, Germany). Stimuli were presented, and responses were recorded using Presentation software (version 16.3; Neurobehavioral Systems Inc., USA). Stimuli from both fMRI experiments were adjusted to the same mean root mean square (rms).

### Vocal sound experiment

The experiment included 60 blocks containing vocal sounds, non-vocal sounds or silence (20 blocks per condition; Belin *et al.*, 2000). Sounds were downloaded from the web site http://vnl.psy.gla.ac.uk/resources.php. Blocks were presented in a randomised order (Figure 1A). Blocks of vocal sounds included speech (e.g. words and foreign language) and non-speech sounds (e.g. laughs and sighs). Blocks of non-vocal stimuli included sounds from the modern environment (e.g. car sounds), nature (e.g. wind), animals (e.g. birdsong) and musical instruments (e.g. saxophone). Participants were instructed to close their eyes and listen attentively. The scanning took approximately 12 min. After scanning, participants wrote down, as accurately as possible, the names of all sounds they remembered hearing during scanning.

### Voice identity recognition experiment

Stimuli consisted of auditory-only two-word sentences spoken by three professional male native German speakers (22, 25 and 26 years old). The sentences were semantically neutral, phonologically and syntactically homogenous [the pronoun ‘er’ (‘he’) and a verb, e.g. ‘Er kauft.’ (‘He buys.’)], and spoken in a neutral manner. All speakers were unfamiliar to the participants. Recordings were made in a soundproofed chamber under the same conditions using the same equipment (condenser microphone, TLM 50; Neumann, Germany; preamplifier, Mic-Amp F-35; Lake People, Germany; soundcard, Power Mac 5; Apple Inc., USA) and the software Sound Studio 3 (Felt Tip Inc., USA) with a 48 000 Hz sampling rate and a resolution of 16 bits. Stimuli were postprocessed and rms adjusted using Matlab (version 7.7; The MathWorks, Inc., USA).

#### Voice identity and speech tasks (fMRI)

The fMRI experiment included two conditions for which we presented exactly the same stimuli: a voice identity and a speech task (Figure 1B). Each condition was presented in 18 blocks (36 blocks in total). At the beginning of each block, participants saw the word ‘speech’ or ‘speaker’ on the screen to inform them about which task to perform (Figure 1C). At the same time, they heard a sentence spoken by one of the three speakers (target). This was followed by a stream of 12 two-word sentences (test sentences) spoken by one of the three speakers. In the voice identity task, participants memorised the target speaker and indicated for each sentence in the ensuing block whether it was spoken by the target speaker or not, independent of the content of the sentence. In the speech task, participants memorised the content of the target sentence and indicated in the ensuing block for each sentence whether it had the same content, independent of the voice identity. Each task included 216 trials (432 trials in total). Each trial was 1.5-sec long. In each trial, one test sentence was presented for approximately 0.9 sec, and the response window was open until the end of the trial. Between blocks, there was a silent period of 18 sec in which a fixation cross was presented on the screen. The sentences within one block were three phonologically similar sentences [e.g. ‘Er sieht’ (‘He sees’), ‘Er siegt’ (‘He wins’), ‘Er singt’ (‘He sings’)]. Within the experiment, each block was presented twice: On one presentation participants performed the voice identity task and the other time the speech task, respectively. Blocks and trials within each block were presented in a randomised order. The number of target items varied between two and four across the blocks and was the same between conditions. All three speakers were presented to the same amount of times as the target speaker (voice identity task) and spoke the same amount of target sentences (speech task). Responses were made via a button box using the target and middle finger of the dominant hand. Total scanning time was approximately 24 min.

Before the voice identity recognition fMRI experiment, participants were familiarised with the three speakers’ voices (speaker familiarisation) and the task (task familiarisation) on a laptop outside the scanner room. All stimuli in the familiarisation phase were presented via headphones (HD 201, Sennheiser, Germany) and were not used during the fMRI experiment.

#### Speaker familiarisation

First, participants were briefly familiarised with the three speakers. They listened to 20 sentences from each of the three speakers organised in blocks (two blocks per speaker, one block containing 10 sentences). Next, pairs of sentences spoken by one or two of the three speakers were presented consecutively and participants indicated whether the sentences were spoken by the same speaker or by two different speakers. Answers were made by pressing a corresponding button on the keyboard. In total, we presented 54 pairs of sentences (18 sentences per speaker). Visual feedback indicating whether the answer was correct (green cross) or wrong (red cross) was provided immediately after each response. We told the participants that this test served to learn the three speaker’s voices for a subsequent voice identity and speech recognition test (voice identity and speech tasks see below).

#### Task familiarisation

After the speaker familiarisation, participants received task instructions for the fMRI experiment and were familiarised with the voice identity and the speech tasks (two practice blocks per condition). The order of the conditions within the task familiarisation was randomised across participants. In order to ensure that all participants understood the task equally well, the task familiarisation was repeated if a participant performed less than 70% correct in the practice trials.

### Image acquisition

Structural T1-weighted and functional images were acquired on a 3T Siemens Magnetom Verio scanner (Siemens, Germany) with a 32-channel head coil for the structural and a 12-channel head coil for the functional images. In the vocal sound experiment, 60 volumes were acquired for each participant. Volumes were acquired at the end of each block (TR 11 s) allowing stimuli presentation without additional noise of the scanner gradients (Belin *et al.*, 2000; Gervais *et al.*, 2004). In the voice identity recognition experiment, 507 volumes were acquired for each participant with a continuous acquisition (TR 2.81 s; von Kriegstein *et al.*, 2003). For details see Supplementary Methods.

### Data analysis

Behavioural data were analysed with PASW Statistics 18.0 (IBM SPSS Statistics, USA). For group comparison, we used analysis of variance (ANOVAs) and independent *t*-tests. We used paired samples *t*-tests for within-group comparisons. All statistical tests were calculated two tailed. Level of significance was defined at *α* = 0.05. MRI data were analysed using standard procedures in Statistical Parametric Mapping (SPM version 8.4667; Wellcome Trust Centre for Neuroimaging, UCL, UK) in a Matlab environment (version 7.11; The MathWorks, Inc., USA). For details see Supplementary Methods.

#### Region of interest

We identified the STS/STG based on probabilistic maps provided in a standard anatomical atlas (Harvard-Oxford cortical structure atlas; Desikan *et al.*, 2006) implemented in FSL (Smith *et al.*, 2004; http://www.fmrib.ox.ac.uk/fsl/fslview) and on peak activation reported in previous studies on vocal sound processing and voice identity recognition (Belin *et al.*, 2000; von Kriegstein *et al.*, 2003; von Kriegstein and Giraud, 2004; von Kriegstein *et al.*, 2005; Blank *et al.*, 2011; Blank *et al.*, 2014) (Supplementary Materials and Methods, ‘Creating masks for ROI analyses’). Previous studies in neurotypical participants have reported responses for the contrast vocal *vs* non-vocal sounds in bilateral STS/STG (Belin *et al.*, 2000). We therefore used bilateral STS/STG maps as the region of interest for the vocal sound experiment. For the contrast voice identity *vs* speech recognition, responses have been reported in anterior and posterior STS/STG of the right hemisphere (von Kriegstein *et al.*, 2003; von Kriegstein and Giraud, 2004; Schall *et al.*, 2015), and both regions are thought to serve different functions in voice identity recognition (Belin and Zatorre, 2003; von Kriegstein *et al.*, 2003; von Kriegstein and Giraud, 2004; Andics *et al.*, 2010; Schall *et al.*, 2015). We therefore used the right anterior and posterior STS/STG maps as the region of interest for the voice identity recognition experiment. The divisions were based on the right STS/STG probabilistic map (Supplementary Materials and Methods; Supplementary Figure S1).

In patients with lesions or neurodegenerative diseases, difficulties in person recognition with both faces and voices have been associated with dysfunction in supramodal regions of the brain (Neuner and Schweinberger, 2000; Gainotti *et al.*, 2003; Hailstone *et al.*, 2011; for review see Gainotti, 2011). Since individuals with high-functioning ASD have difficulties with both voice and face recognition (Schelinski *et al.*, in press), we performed an exploratory region of interest (ROI) analysis in supramodal brain regions, i.e. the right precuneus, left middle temporal gyrus and the right medial temporal pole (see Supplementary Materials and Methods).

### Significance thresholds for fMRI data

Effects were considered as significant at *P* < 0.05 family wise error (FWE) corrected for the ROI. We considered effects for which we did not have a priori hypotheses significant at *P < *0.05 FWE corrected for the whole brain. For information purposes only, all clusters at a threshold of *P* = 0.001 uncorrected are reported in Supplementary Table S3.

## Results

### Behavioural results

**Fig. 2.**
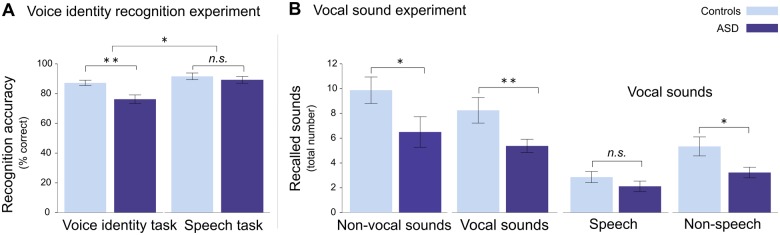
Behavioural results from the two fMRI experiments (see also Table 2 and Supplementary Table S3). (A) Performance accuracy in the voice identity recognition experiment: The ASD group performed significantly worse than the control group in the voice identity task. There were no significant differences between the ASD and the control group in the speech task. (B) Total amount of recalled sounds after the vocal sound experiment. The ASD group recalled significantly less non-vocal and vocal sounds than the control group. The vocal sound condition contained both speech and non-speech sounds. The ASD group recalled a comparable number of speech sounds but less non-speech sounds as compared to the control group. Error bars represent± 1 SE; *P < 0.05; **P < 0.005; n.s. not significant.

During the fMRI voice identity recognition experiment, participants performed tasks on voice identity and speech recognition with recently learned voices. The ASD group was significantly impaired in voice identity recognition, but not in speech recognition. This was revealed by a repeated-measures ANOVA with the between-subjects factor ‘group’ (controls, ASD) and the within-subject factor ‘task’ (voice identity and speech) and *post hoc t*-tests. The ANOVA revealed a significant interaction between task and group (*F*(1,30) = 5.549, *P* = 0.025). There was also a main effect of task (*F*(1,30) = 22.563, *P* < 0.001) and group (*F*(1,30) = 5.787, *P* = 0.023) (Figure 2A; Table 2). There was a significant group difference in the voice identity task (*t*(30) = 3.228, *P* = 0.003) but not in the speech task (*t*(30) = 0.723, *P* = 0.475), indicating that the ASD group performed significantly worse than controls in voice identity but not in speech recognition. The ASD group but not the control group performed worse in the voice identity task as compared to the speech task (ASD group: *t*(15) = −4.851, *P* < 0.001; control group: *t*(15) = −1.758, *P* = 0.099). This indicates that for controls, the level of difficulty was roughly matched between the tasks. Also the results of the vocal sound experiment indicated that the voice identity recognition difficulties dissociate from intact speech recognition abilities in high-functioning ASD: The ASD group recalled significantly less non-speech vocal sounds as compared to the control group after the fMRI scan (*t*(30) = 2.396, *P* = 0.023). In contrast, there were no significant group differences in the amount of recalled speech vocal sounds (*t*(30) = 1.202, *P* = 0.239; Figure 2B; Table 2).

**Table 2.** Summary of average scores for all experiments. Scores are summarised as average over group with standard deviation (SD) and p- values from independent t- tests. All analyses include data from 16 ASD participants and their 16 pairwise matched control participants

	ASD		Controls		
Voice tests	*M*	*SD*	*M*	*SD*	*P*
Voice identity recognition experiment (recognition accuracy %)					
Voice identity task	76.36	11.61	87.36	7.15	0.003*
Speech task	89.41	9.28	91.75	9.06	0.475
Vocal sound experiment (number of recalled sounds)					
Total	11.88	6.25	18.13	7.20	0.014*
Vocal sounds	5.38	2.16	8.25	4.12	0.019*
Speech	2.13	1.71	2.88	1.82	0.239
Non-speech	3.25	1.73	5.38	3.10	0.023*
Non-vocal sounds	6.50	4.93	9.88	4.27	0.047*
Nature	1.19	1.64	1.44	1.75	0.680
Animals	2.31	2.70	3.31	2.21	0.261
Modern environment	2.31	1.30	4.31	2.12	0.003*
Musical instruments	0.69	0.95	0.81	0.98	0.716

*Significant group differences (*P* < 0.05).

### fMRI results

#### Vocal sound experiment

**Fig. 3.**
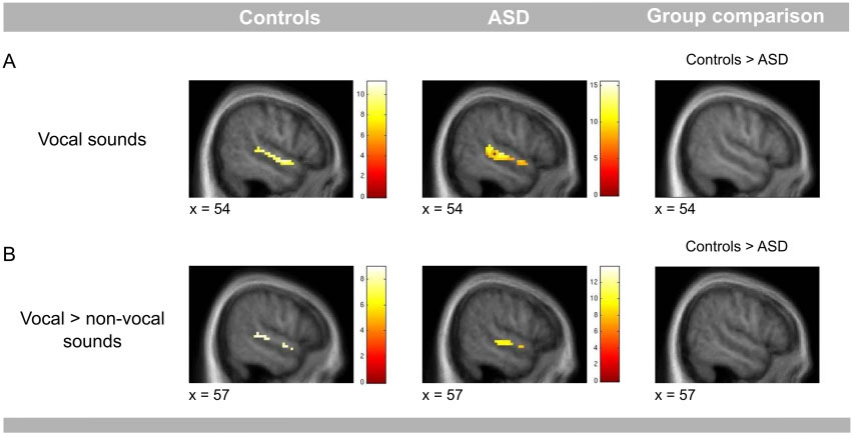
Vocal sound experiment. (A) Contrast vocal sounds > silence baseline. The control group as well as the ASD group showed BOLD responses along the STS/STG when listening to vocal sounds. The figure displays results for the right STS/STG, for the left STS/STG see Supplementary Table S2. (B) Contrast vocal > non-vocal sounds. Both groups showed enhanced BOLD responses in the right STS/STG when listening to vocal, compared to non-vocal, sounds. The results are displayed at the threshold of *P* = 0.05 FWE corrected for the right STS/STG. They are overlaid onto a group specific average of normalised T1- weighted structural images. Colour bars represent *t*-values.

Listening to vocal sounds compared to the silence baseline (Figure 3A) and listening to vocal, compared to non-vocal sounds (Figure 3B) elicited a significantly higher BOLD response along the right and left STS/STG in both groups. There was no significant difference in the responses of the control group and the ASD group in this region (Supplementary Table S2). Because a previous study reported a lack of TVA responses in four of five tested ASD participants at *P* < 0.001 uncorrected, we also checked individual responses in bilateral STS/STG. We found that 15 out of 16 participants in the ASD group had bilateral TVA responses at *P* < 0.001 uncorrected when listening to vocal as compared to non-vocal, sounds. The remaining ASD participant had TVA responses at *P* < 0.002. All 16 control participants had TVA response in the right hemisphere (*P* < 0.001 uncorrected) and 15 out of 16 controls in the left hemisphere (one control participant at *P* < 0.008).

#### Voice identity recognition experiment

**Fig. 4.**
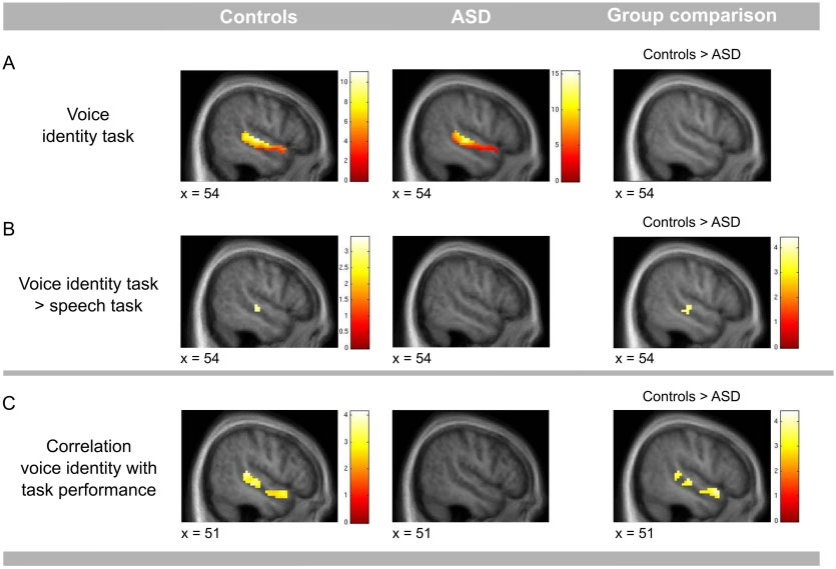
Voice identity recognition experiment. (A) Contrast voice identity task > silence baseline. The control group as well as the ASD group showed BOLD responses along the right STS/STG when the task was to recognise voice identity. (B) Contrast voice identity task > speech task. The control group showed greater BOLD responses when recognising voice identity compared to when recognising speech. In the right posterior STS/STG these responses were higher for the control group as compared to the ASD group. (C) In the control, but not in the ASD group, responses in the right STS/STG to voice identity recognition correlated positively with performance in voice identity recognition. This correlation was stronger in the anterior STS/STG in the control group as compared to the ASD group. Results are presented for the right STS/STG and overlaid onto a group specific average image of normalised T1- weighted structural images. The results are significant at *P* = 0.05 FWE corrected for the ROI. For display purposes only the threshold of *P* = 0.01 uncorrected was used. Colour bars represent *t*-values.

Both groups had significant BOLD responses in the right anterior and posterior STS/STG when performing the voice identity task in comparison to the silence baseline. There were no significant differences between the groups (Figure 4A; Table 3). For the voice identity task, as compared to the speech task, we found a BOLD response in the right posterior STS/STG for the control group only (Figure 4B; Table 3). In the ASD group, no responses were present for this contrast (voice identity task > speech task), even at a lenient statistical threshold (*P* < 0.01 uncorrected). The difference between groups was statistically significant in the right posterior STS/STG (task × group interaction: [(speaker identity task/controls > speech task/controls) > (speaker identity task/ASD > speech task/ASD)]; Figure 4B; Table 3; Supplementary Figure S2). This group difference was also significant when we applied a Bonferroni correction for two ROIs (*P* < 0.025 FWE corrected).The group difference could not be explained by a difference in BOLD responses in the speech task; there was no simple main effect for the speech task (Supplementary Table S3) and also the signal change for each condition separately (Supplementary Figure S2) indicated a similar level of BOLD responses for the speech task in the control and the ASD group. Within the control group, behavioural performance in the voice identity task correlated significantly with BOLD responses for the voice identity task in the right anterior and posterior STS/STG (Figure 4C; Table 3). In contrast, in the ASD group, even at a lenient statistical threshold (*P* < 0.01 uncorrected), there was no such correlation in none of the two ROIs. The correlation was significantly higher in the control group than in the ASD group in the anterior part of the right STS/STG (Bonferroni corrected). This finding suggests that the ASD group engaged the STS/STG voice-sensitive regions differently than controls, and that voice recognition performance in the ASD group does not rely on the integrity of voice-sensitive areas to the same degree as it does in controls.

**Table 3.** Coordinates for significant BOLD-responses in the voice recognition experiment (*p* < 0.05 FWE- corrected at peak level for the region of interest)

	Voice identity task									
	Controls					ASD				
Right STS/STG	x	y	z	*Z*	Cluster size	x	y	z	*Z*	Cluster size
Anterior	54	11	−11	3.74	49	57	8	−5	3.11	81
Posterior	54	−19	1	5.68	342	66	−25	10	6.42	342
	
	Controls > ASD					ASD > Controls				
Anterior/posterior			–					–		

	Voice identity task > speech task									
	Controls					ASD				
Right STS/STG	x	y	z	*Z*	Cluster size	x	y	z	*Z*	Cluster size

Anterior	–					–				
Posterior	54	−22	−2	3.35	332	–				
	
	Controls > ASD					ASD > controls				
Anterior			–			–				
Posterior	51	−19	−2	3.63	332	–				

	Correlation voice identity with task performance									
	Controls					ASD				
Right STS/STG	x	y	z	*Z*	Cluster size	x	y	z	*Z*	Cluster size

Anterior	54	11	−17	3.31	49			–		
Posterior	48	−34	7	3.63	332			–		
	
	Controls > ASD					ASD > controls				
Anterior	54	11	−14	3.51	49			–		
Posterior	–						–	

Coordinates represent local activation maxima in MNI space (in mm). Cluster size represents the number of voxels within a cluster.

There were no significant correlations between behavioural performance and BOLD responses for the contrast voice identity task *vs* speech task.

We additionally checked whether the significant group differences for the voice identity *vs* speech task in the voice identity recognition experiment were located in regions that are sensitive to the contrast vocal *vs* non-vocal sounds in the vocal sound experiment. To do that, we defined a STG/STS-ROI based on a conjunction of the contrast vocal > non-vocal sounds in the ASD group and vocal > non-vocal sounds in controls (threshold for both contrasts at *P* = 0.01). We found significantly higher BOLD response for the control group as compared to the ASD group in the posterior STS/STG for the voice identity task, as compared to the speech task (peak at *x* = 51, *y* = −19, *z* = −2; *P = *0.026 FWE corrected) and in the anterior STS/STG for the correlation between the behavioural performance in the voice identity task and BOLD response for the voice identity task (peak at *x* = 54, *y* = 11, *z* = −14; *P* < 0.028 FWE corrected) (Supplementary Figure S3).

#### ROI analyses in supramodal brain regions

In the control group, the right precuneus responded in the contrast voice identity task > speech task. There was also a positive correlation of responses in the voice identity task with task performance in the voice identity task (Supplementary Table S5). There were no significant responses in the right precuneus within the ASD group. The control group showed significantly higher responses as compared to the ASD group in the right precuneus for the voice identity task and the contrast voice identity task > speech task (Supplementary Table S5). There was no significant response in either group and no differences between groups for the left middle temporal gyrus and right medial temporal pole.

#### Head motion during fMRI

The ASD and the control group did not differ significantly in the average amount of head movements (all *P*s > 0.1; see Supplementary Table S6).

## Discussion

Our study on voice processing in high-functioning ASD revealed three key findings. First, we found typical responses in voice-sensitive cortices in the STS/STG (temporal voice areas, TVA) in adults with high-functioning ASD and neurotypically developed controls, for passive listening to vocal sounds in contrast to non-vocal sounds. Second, for voice identity recognition, in contrast to speech recognition, a part of the voice-sensitive cortices, i.e. the posterior STS/STG responded less in ASD than in controls. Third, the anterior STS/STG did not correlate with voice recognition performance in ASD but in controls. These findings fundamentally advance our understanding of the voice recognition deficit in ASD, because they (i) reveal for the first time brain response profiles of voice processing in a group of well-matched ASD and control groups and (ii) allow for assessing the relation between voice recognition behaviour and neuroimaging data in ASD. The results give insight into the behavioural relevance of voice-sensitive regions in the STS/STG and suggest that the voice identity recognition deficit in adults with high-functioning ASD is based on a selective dysfunction of right hemispheric STS/STG voice-sensitive cortices.

The behavioural and neuroimaging findings of the present study converge to show that people with high-functioning ASD have a relatively selective deficit in voice processing. The behavioural results confirmed earlier findings of voice recognition impairments in ASD (Boucher *et al.*, 1998; Schelinski *et al.*, 2014). Voice recognition deficits have been found in children with ASD and in adults with high-functioning ASD. Our findings replicated previous findings that voice identity and speech recognition are differentially affected in high-functioning ASD (Schelinski *et al.*, 2014). In addition, in a previous study on the same ASD group as in the present study, the ASD group had particular difficulties with discriminating and learning unfamiliar voices, while recognising famous voices was not significantly different from controls (Schelinski *et al.*, in press). The neuroimaging findings of the present study fit this relatively selective behavioural deficit well. They show that people with high-functioning ASD have typical responses to vocal sounds in TVAs, indicating that voice processing in ASD is not impaired in general. The dysfunction only becomes apparent during voice recognition in parts of the voice-sensitive cortices.

The typical responses in voice-sensitive STS/STG in the ASD group to vocal sounds contrasts a previous report of a lack of voice-sensitive STS/STG responses to vocal sounds in four out of five ASD participants (Gervais *et al.*, 2004). We speculate that differences in the sample characteristics might explain the TVA response differences in the two studies. In the study by Gervais *et al.* (2004), participants were matched on age and gender and a measure of verbal fluency did not show significant differences between the groups. It is unclear whether the general IQ or the verbal IQ was matched across groups. In our study, each control participant was matched to the respective ASD participant based on age, gender, handedness, and full-scale IQ and also the verbal IQ was comparable between the two groups. A potential difference between the ASD and the control participants in general cognitive abilities or a potential difference in verbal abilities might have led to the absent TVA responses in the ASD in contrast to the control group in the .study by Gervais *et al.*(2004). Alternatively, differences in cognitive and potentially also verbal abilities between the samples of the Gervais *et al.* (2004) and our study might explain the different results. Against the influence of the general IQ speaks that even the four participants in our study that were closest in general IQ scores (85–100) to the study by Gervais *et al.* (2004) (four participants 85–95, 1 at 50), had TVA responses. The present study does, however, not permit to adjudicate between the different explanations, but it highlights the importance of a careful match between ASD and control groups on cognitive and particularly verbal abilities when investigating voice recognition.

A neuroimaging study in neurotypical participants has shown that recognising unfamiliar voices (in contrast to recognising speech) recruits a posterior STS/STG region (MNI-coordinate: *x* = 48, *y* = −21, *z *= −12; von Kriegstein and Giraud, 2004) to a higher extent than recognising personally familiar voices (in contrast to recognising speech). In the present study, it is this posterior STS/STG region that we found to be less responsive during voice recognition (in contrast to speech recognition) in ASD than in controls (MNI-coordinate: *x* = 51, *y* = −19, *z* = −2). Several studies in typically developed individuals have shown that the posterior part of the right STS/STG is associated with the processing of complex spectrotemporal voice features and these features might be of higher relevance for unfamiliar than for familiar voices (von Kriegstein and Giraud, 2004; Warren *et al.*, 2006; von Kriegstein *et al.*, 2007; Andics *et al.*, 2010; von Kriegstein *et al.*, 2010; Blank *et al.*, 2014). We therefore speculate that people with high-functioning ASD have difficulties in analysing and integrating the acoustic voice features and that this is the cause for their voice recognition impairments for unfamiliar voices. Such a process might be particularly important for learning novel voices in ASD, whereas recognising familiar (i.e. famous) voices might rely on a partially different mechanism. In this view, the lack of correlation with voice recognition performance in the anterior STS/STG would be a result of dysfunctional processing in the posterior STS/STG.

In addition to differences in voice-sensitive brain areas, we found group differences between the ASD group and the control group in the precuneus, a typical supramodal person recognition area (for meta-analysis see Blank *et al.*, 2014). The precuneus and adjacent regions (i.e. the posterior cingulate and cuneus) have also been associated with atypical face processing in ASD (Pierce *et al.*, 2004; Kleinhans *et al.*, 2008; Domes *et al.*, 2013). This might suggest that the person recognition deficit in ASD is of a supramodal nature. In accordance with this, a correlation between face and voice identity recognition abilities was present in the ASD group but not in the control group (Schelinski *et al.*, in press). However, there are several findings that speak against a supramodal aetiology for the person recognition deficit in ASD. First, the behavioural pattern that we found in the voice processing tests in the same ASD group suggested impairments at processing voice information even at a perceptual level, i.e. the discrimination of voices as same or different and vocal pitch discrimination (Schelinski *et al.*, in press). Second, the perception of famous voices and the association with names or semantic information was relatively intact in the same ASD group. Third, there were response differences in the posterior STS/STG that fit with a perceptual deficit in voice processing. Fourth, several studies on face recognition in ASD have also found not only supramodal brain regions impaired, but a processing impairment in the fusiform face area (FFA; e.g. Schultz *et al.*, 2000; Pierce *et al.*, 2001; Domes *et al.*, 2013), a brain region that is associated with face perception and identity processing (Eger *et al.*, 2004; von Kriegstein *et al.*, 2008; Hermann *et al.*, 2015; for review see Bernstein and Yovel, 2015). We therefore speculate that a dysfunctional precuneus is not the primary cause for the person recognition deficits for voices and faces in ASD.

The results of the present study are partly in agreement with current voice recognition models (for reviews see Belin *et al.*, 2004; Blank *et al.*, 2014). The finding of spared speech recognition together with dysfunctional voice recognition mechanisms in the ASD group is in line with a central assumption of models on human communication (Belin *et al.*, 2004; von Kriegstein *et al.*, 2008): Although partially overlapping (e.g. Lachs and Pisoni, 2004; Perrachione and Wong, 2007; von Kriegstein *et al.*, 2010; Kreitewolf *et al.*, 2014), speech and voice identity recognition are distinguishable abilities that are processed relatively independently in the human brain (Belin and Zatorre, 2003; Formisano *et al.*, 2008). However, a distinction between processing of familiar and unfamiliar voices is not yet featured in voice or person recognition models (Ellis *et al.*, 1997; Belin *et al.*, 2004; Blank *et al.*, 2014). A relatively selective deficit in unfamiliar voice recognition (Schelinski *et al.*, in press) and a predominant involvement of the posterior STS/STG in unfamiliar voice recognition as implicated by the present and previous neuroimaging results (von Kriegstein and Giraud, 2004) might indicate that neuroscientific models of voice recognition (Belin *et al.*, 2004; Blank *et al.*, 2014) need to be amended to incorporate potential differences in the processing mechanisms for familiar and unfamiliar voices at the perceptual level.

Investigating voice processing in ASD provides not only clinical evidence to inform general models of human communication but also insight into the characteristics of the life-long communication difficulties in ASD. Voice recognition is an evolutionary conserved process (Petkov *et al.*, 2009; Proops *et al.*, 2009; Andics *et al.*, 2014) and specific neural responses to voices can be already observed very early in infancy (Grossmann *et al.*, 2010; Blasi *et al.*, 2011). There is evidence that atypical voice perception in ASD appears already early in life (Klin, 1991). Life-long perceptual impairments with vocal information might significantly exacerbate difficulties with social interactions—a core feature of ASD.

## Supplementary Material

Supplementary Data
