# Supplementary material for: Temporal voice areas exist in autism spectrum disorder but are dysfunctional for voice identity recognition
Source: Soc Cogn Affect Neurosci. 2016 Jun 30;11(11):1812–22. doi: 10.1093/scan/nsw089 (PMC5091681; doi:10.1093/scan/nsw089)
Supplement: Supplementary Data [file supp_11_11_1812__index.html]

Temporal voice areas exist in autism spectrum disorder but are dysfunctional for voice identity recognition — Temporal voice areas exist in autism spectrum disorder but are dysfunctional for voice identity recognition — Supplementary Data 

# Temporal voice areas exist in autism spectrum disorder but are dysfunctional for voice identity recognition

## Supplementary Data

files

- Supplementary Data - docx file
