# Supplementary material for: Temporal voice areas exist in autism spectrum disorder but are dysfunctional for voice identity recognition
Source: Soc Cogn Affect Neurosci. 2016 Jun 30;11(11):1812–22. doi: 10.1093/scan/nsw089 (PMC5091681; doi:10.1093/scan/nsw089)
Supplement: Supplementary Data [file supp_nsw089_scan-16-079-File010.docx]

**Supplementary Materials and Methods**

*Image acquisition*

*Structural MRI.* Anatomical images were acquired using a 32-channel head coil and a T1-weighted 3D magnetisation-prepared rapid gradient echo (MPRAGE) sequence (Mugler and Brookeman, 1990) (TR = 2300 ms; TE = 2.98 ms; TI = 900 ms; flip angle = 9°; FOV = 256 mm x 240 mm; voxel size = 1 mm^3^ (isotropic resolution) 176 sagittal slices) with nonselective excitation and linear phase encoding. Magnetisation preparation consisted of a nonselective inversion pulse. For one participant we used a 12-channel head coil (with an identical scanning protocol), as the 32-channel head coil was too tight for the participants head size. Scanning time for the structural scan was 9 min 14 s.

*Functional MRI.* fMRI images were acquired using a gradient-echo EPI (echo planar imaging) pulse sequence (TE = 30 ms; flip angle = 90°; FoV = 192 mm x 192 mm; 2 mm slice thickness; interslice gap = 1mm; voxel size = 3 mm x 3 mm x 2 mm; 42 axial slices; acquisition bandwidth = 1954 Hz; whole brain coverage; ascending acquisition). A pair of 2D gradient echo images with different echo times (TE1/TE2 = 4.92 ms/7.38 ms) was obtained for B0 field mapping (Jezzard and Balaban, 1995). These images were measured at the same slice locations and as in the fMRI acquisition. Voxel resolution and images size were the same. Scanning parameters were: TR=488 ms, flip angle 60°, pixel bandwidth=327 Hz/pixel. Images were acquired AC-PC oriented.

**Data analysis**

*Behavioural data.* For the voice identity recognition experiment, we used the average percentage of correct responses as the dependent variable for analyses. For the analyses of the behavioural data in the vocal sound experiment we used the absolute number of recalled sounds for each sound category. The numbers of sounds for each category presented in the experiment are unequal. Thus, we report direct group comparisons i.e. independent *t*-test for each sound category but did not perform repeated measures ANOVAs for the total numbers of recalled sounds.

*MRI data.* MRI data were analysed using Statistical Parametric Mapping (SPM version 8.4667; Wellcome Trust Centre for Neuroimaging, UCL, UK) in a Matlab environment (version 7.11, The MathWorks, Inc., USA). For preprocessing, images were realigned and unwarped. Anatomical scans were coregistered to the mean of the functional scans. Images were normalised to the Montreal Neurological Institute (MNI) standard stereotactic space and spatially smoothed with a Gaussian kernel of 8 mm full width at half maximum. For all analyses, statistical parametric maps were generated by modelling the evoked hemodynamic response for the different conditions as boxcar functions convolved with a synthetic hemodynamic response function using the general linear model (Friston *et al.*, 2007) (high-pass filter 128 s). We modelled the conditions ‘vocal’ and ‘non-vocal’ in the vocal sound experiment. In the voice identity recognition experiment we modelled the conditions ‘voice identity task’, ‘speech task’, and ‘instruction’ at the first level*.* To account for group differences in performance in the voice identity recognition experiment, we included individual behavioural performance scores (percent correct) as a covariate of no interest at the second level analyses: For the group comparison of the contrast ‘voice identity > silence baseline’ the covariate of no interest was the individual performance in the voice identity task. For within and between group analyses for the contrast ‘voice identity’ > ‘speech’ we used the performance difference between speech and voice identity task as a covariate of no interest.

For both experiments, we performed one-sample *t*-tests across the single-subject contrast images for within group analyses. For between group analyses we used two-sample *t*-tests comparing the means of the single-subject contrast images from both groups.

*Creating masks for ROI analyses*

*STS/STG.* There are no standard maps available for voice-sensitive regions along the STS/STG. For our ROI analyses we extracted the following probabilistic maps provided by a standard anatomical atlas (Harvard-Oxford cortical structure atlas; Desikan *et al.*, 2006) implemented in FSL (Smith *et al.*, 2004; http://www.fmrib.ox.ac.uk/fsl/fslview): right temporal pole, right anterior division of the superior temporal gyrus, and right posterior division of the superior temporal gyrus. We set the threshold for extraction to 10 (i.e. overlap of at least 10% of the individual maps used to generate the ROIs provided in the atlas), because at this threshold the map covered most previously reported peaks of activation for voice processing in the temporal lobe (Belin *et al.*, 2000; von Kriegstein *et al.*, 2003; von Kriegstein and Giraud, 2004; von Kriegstein *et al.*, 2005; Blank *et al.*, 2011; Blank *et al.*, 2014; 18 from 23 peak activations were included; Supplementary Figure 1). The probabilistic maps provided in the atlas and the peak responses reported in previous studies were all based on group level analyses. To restrict the temporal pole map to the STS/STG, we cut the map according to the following criteria: the medial boundary was defined by the medial extend of the superior temporal sulcus, the inferior boundary by the lower bank of the superior temporal sulcus, and the boundary at the top was defined by the Sylvian fissure. The final map of the voice-sensitive STS/STG was a union of the posterior and anterior STS/STG division provided by the anatomical atlas and the modified temporal pole map and included 536 voxels. The left STS/STG was created by flipping this map to the left hemisphere. For the ROI analyses in the right anterior and posterior STS/STG we used the modified temporal pole map for the anterior STS/STG ROI, and the posterior division minus the anterior division of the superior temporal gyrus for the posterior STS/STG. To assure correspondence in orientation and resolution with the functional data, all masks were co-registered to functional images from our experiment.

*Supramodal brain regions*. For ROI analyses in supramodal brain regions, we created 10 mm spheres around MNI coordinates of supramodal brain regions identified and provided in a meta-analysis by Blank et al., 2014 (MNI coordinates (x,y,z) in mm: right precuneus = 5, -56, 26, left middle temporal gyrus = -53, -2, -30, and right medial temporal pole = 51, 15, -32). Effects were considered significant at p < 0.05 FWE corrected for the ROI and Bonferroni-corrected for the three ROIs (*p* < 0.016 FWE corrected).

**Supplementary Figures**


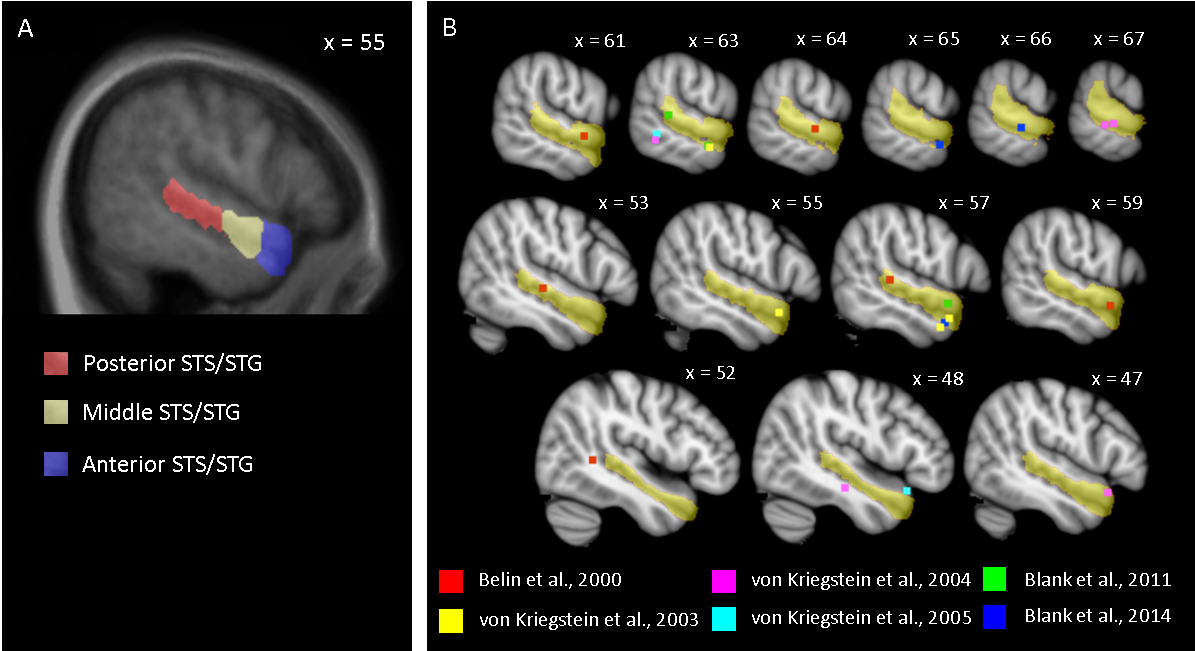


Supplementary Figure 1

STS/STG maps used for ROI analyses. *(A)* Subdivisions of the STS/STG maps. We used the combined STS/STG maps of both hemispheres for the vocal sound experiment and the anterior and posterior STS/STG maps of the right hemisphere for ROI analyses in the voice identity recognition experiment. *(B)* The STS/STG maps covered most previously reported peaks of responses for voice processing in the temporal lobe- 18 from 23 peaks were included.


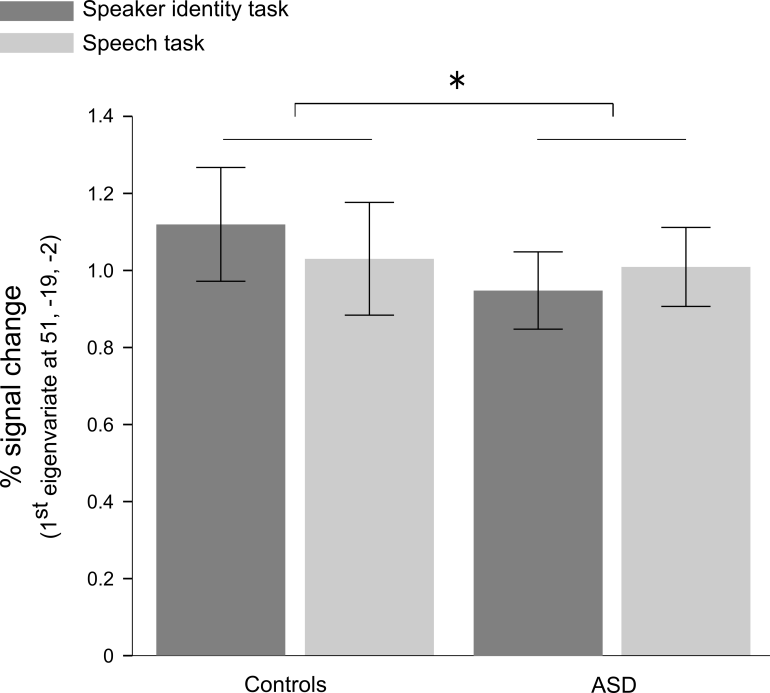


Supplementary Figure 2

Mean 1^st^ eigenvariates extracted at the statistical maximum (MNI-coordinate: x = 51, y = -19, z = -2) of the interaction between group (controls, ASD) and task (speaker identity task, speech task). Error bars represent +/- 1 SE. * indicates the significant interaction effect between group and task for display purposes only.


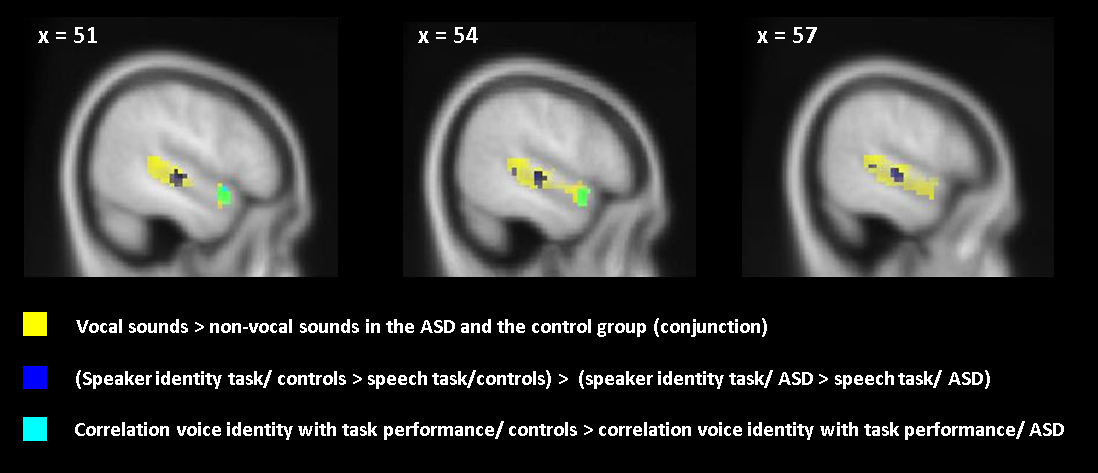


Supplementary Figure 3

fMRI results of the voice identity recognition experiment in relation to the BOLD responses found in the vocal sound experiment along the right STS/STG. Yellow: region of interest as defined by the vocal sound experiment. Blue: BOLD responses that were lower in the ASD group in contrast to the control group for the voice identity task in contrast to the speech task. Displayed for visualisation at *p* = 0.01 uncorrected. Cyan: Lower correlation between BOLD responses in the voice identity task and voice identity task performance in the ASD group as compared to the control group. Displayed for visualization at *p* = 0.01 uncorrected.

**Supplementary Tables**

Supplementary Table 1

Overview of diagnostic scores in the ASD group.

| Diagnostic test | | *M* | *SD* |
| --- | --- | --- | --- |
| **Participants as informant** | | | |
| Questionnaire | AQ^a^ (*n* = 16) | 39.81 | 6.61 |
|  |  |  |  |
| Interview | ADOS^b^ (*n* = 15) |  |  |
|  | Social Interaction & Communication | 11.00 | 2.78 |
|  | Social Interaction | 7.20 | 1.97 |
|  | Communication | 3.80 | 1.27 |
| **Parents as informant** | | | |
| Questionnaire | SCQ^c^ (*n* = 9) | 20.33 | 5.70 |
|  |  |  |  |
| Interview | ADI-R^d^ (*n* = 9) |  |  |
|  | Social Interaction & Communication | 36.22 | 8.04 |
|  | Social Interaction | 21.11 | 5.09 |
|  | Communication | 13.89 | 4.37 |

*M* = mean; *SD* = standard deviation.

^a^ AQ = Autism Spectrum Quotient. A total score of 32+ is suggested as a useful cut-off for distinguishing individuals who have clinically relevant levels of traits associated with autism spectrum (Baron-Cohen *et al.*, 2001).

^b^ ADOS = Autism Diagnostic Observation Schedule (Lord *et al.*, 2000; German version: Rühl *et al.*, 2004).

^c^ SCQ = Social Communication Questionnaire (Rutter *et al.*, 2003; German version: Bölte and Poustka, 2006).

^d^ ADI-R = Autism Diagnostic Interview- Revised (Lord *et al.*, 1994; German version: Bölte *et al.*, 2003).

Supplementary Table 2

Coordinates for BOLD-responses in the vocal sound experiment (*p* < .05 FWE- corrected at peak- level for regions of interest). For the contrast ASD > Controls we did not have a specific hypothesis and therefore report this contrast for information purposes only.

|  | **Vocal sounds** | | | | | | | | | | |
| --- | --- | --- | --- | --- | --- | --- | --- | --- | --- | --- | --- |
|  | Controls | | | | |  | ASD | | | | |
|  | x | y | z | *Z* | cluster  size |  | x | y | z | *Z* | cluster  size |
| right STS/STG | 57 | 2 | -8 | 5.72 | 570 |  | 45 | -22 | -2 | 6.44 | 576 |
| left STS/STG | -63 | -19 | 1 | 5.96 | 587 |  | -57 | -28 | 4 | 6.16 | 592 |
|  | Controls > ASD | | | | | | ASD > Controls | | | | |
| right STS/STG | **-** | | | | | | **-** | | | | |
| left STS/STG | **-** | | | | | | **-** | | | | |
|  | | | | | | | | | | | |
|  | **Vocal sounds > non-vocal sounds** | | | | | | | | | | |
|  | Controls | | | | |  | ASD | | | | |
|  | x | y | z | *Z* | cluster  size |  | x | y | z | *Z* | cluster  size |
| right STS/STG | 60 | -4 | -8 | 5.19 | 570 |  | 63 | -16 | -2 | 6.19 | 576 |
| left STS/STG | -63 | -22 | -2 | 5.27 | 587 |  | -60 | -34 | 4 | 6.00 | 592 |
|  | Controls > ASD | | | | | | ASD > Controls | | | | |
| right STS/STG | **-** | | | | | | **-** | | | | |
| left STS/STG | **-** | | | | | | -60 | -31 | 10 | 3.90 | 562 |

Coordinates represent local activation maxima in MNI space (in mm). Cluster size represents the number of voxels within a cluster. STS/STG = Superior Temporal Sulcus and Gyrus.

Supplementary Table 3

Overview of BOLD-response maxima for the main contrasts in the vocal sound experiment and the voice identity recognition experiment for the control and the ASD group (at *p* < 0.001 uncorrected) for information purposes only.

| **Vocal sound experiment** | | | | | | | | | | | | | | | | |
| --- | --- | --- | --- | --- | --- | --- | --- | --- | --- | --- | --- | --- | --- | --- | --- | --- |
|  | | Vocal sounds | | | | | | | | | | | | | | |
|  | | Controls | | | | | | | | ASD | | | | | | |
| Region | | x | | y | z | *Z* | | cluster  size | | x | y | | z | | *Z* | cluster  size |
| STS/STG | r | 51 | | -13 | 4 | 6.06 | | 1196 ^a^ | | 39 | -25 | | 10 | | 6.60 | 1807 ^a^ |
|  | l | -63 | | -19 | 1 | 5.96 | | 1325 | | -36 | -34 | | 10 | | 6.95 | 1729 ^b^ |
| Brain-Stem |  | 0 | | -34 | -2 | 5.05 | | 221 | | 0 | -37 | | -2 | | 4.82 | 242 |
| Cerebellum | l | -15 | | -79 | -44 | 4.55 | | 87 | | -6 | -37 | | -41 | | 4.91 | 75 |
|  |  |  | |  |  |  | |  | | -15 | -76 | | -47 | | 4.87 | 206 |
|  |  |  | |  |  |  | |  | | -42 | -64 | | -29 | | 4.08 | 36 |
|  | r | 21 | | -79 | -38 | 4.45 | | 84 | | 15 | -79 | | -41 | | 5.29 | 169 |
|  |  |  | |  |  |  | |  | | 24 | -61 | | -23 | | 3.82 | 10 |
| Frontal Operculum | l |  | |  |  |  | |  | | -39 | 26 | | 4 | | 4.57 | 73 |
| Amygdala | r |  | |  |  |  | |  | | 21 | -7 | | -14 | | 4.42 | 36 |
| Precentral Gyrus | r |  | |  |  |  | |  | | 54 | -1 | | 46 | | 4.35 | 35 |
| Thalamus | r |  | |  |  |  | |  | | 15 | -13 | | 7 | | 3.78 | 15 |
| SMA | r |  | |  |  |  | |  | | 6 | 5 | | 64 | | 3.70 | 28 |
|  | Controls > ASD | | | | | | | | | | | | | | | |
|  |  | | x | | | | y | | z | Z | | cluster  size | |  | | |
|  | - | | | | | | | | | | | | | | | |
|  | ASD > Controls | | | | | | | | | | | | | | | |
| Cerebellum | r | | 21 | | | | -49 | | -44 | 3.87 | | 21 | |  | | |

|  | | Vocal sounds > non-vocal sounds | | | | | | | | | | | | | |
| --- | --- | --- | --- | --- | --- | --- | --- | --- | --- | --- | --- | --- | --- | --- | --- |
|  | | Controls | | | | | | | ASD | | | | | | |
| Region | | x | y | z | *Z* | | cluster  size | | x | y | | z | | *Z* | cluster  size |
| STS/STG | l | -63 | -22 | -2 | 5.27 | | 498 | | -60 | -34 | | 4 | | 6.00 | 839 |
|  | r | 60 | -4 | -8 | 5.19 | | 581 | | 63 | -16 | | -2 | | 6.19 | 705 |
| Precentral Gyrus | r |  |  |  |  | |  | | 54 | 5 | | 43 | | 4.16 | 28 |
|  | l |  |  |  |  | |  | | -48 | -10 | | 40 | | 3.87 | 21 |
| Frontal Operculum | l |  |  |  |  | |  | | -39 | 23 | | 4 | | 4.13 | 37 |
| Central Operculum | l |  |  |  |  | |  | | -36 | 8 | | 10 | | 4.02 | 18 |
| Cerebellum | l | -18 | -76 | -35 | 4.10 | | 26 | |  |  | |  | |  |  |
|  | r |  |  |  |  | |  | | 27 | -61 | | -23 | | 4.09 | 9 |
|  |  |  |  |  |  | |  | | 18 | -76 | | -41 | | 4.06 | 10 |
| SMA | r |  |  |  |  | |  | | 6 | 2 | | 67 | | 3.96 | 40 |
| Amygdala | l |  |  |  |  | |  | | -18 | -7 | | -14 | | 3.94 | 13 |
| Insula | l |  |  |  |  | |  | | -36 | 2 | | -17 | | 3.74 | 10 |
|  | Controls > ASD  (≙ ASD > Controls for non-vocal sounds > vocal sounds) | | | | | | | | | | | | | | |
|  |  | x | | | | y | | z | Z | | cluster  size | |  | | |
|  | - | | | | | | | | | | | | | | |

|  | | Non-vocal sounds | | | | | | | | | | | | | | |
| --- | --- | --- | --- | --- | --- | --- | --- | --- | --- | --- | --- | --- | --- | --- | --- | --- |
|  | | Controls | | | | | | | | ASD | | | | | | |
| Region | | x | | Y | z | *Z* | | cluster  size | | x | y | | z | | *Z* | cluster  size |
| STS/STG | r | 48 | | -13 | 7 | 5.81 | | 996 ^a^ | | 51 | -22 | | 7 | | 6.55 | 1524 ^a^ |
|  | l | -42 | | -28 | 13 | 5.75 | | 1049 ^a^ | | -36 | -34 | | 13 | | 6.80 | 1459 ^b^ |
| Brain-Stem | r | 0 | | -40 | -5 | 5.57 | | 190 | | 3 | -40 | | -11 | | 5.02 | 146 |
|  |  |  | |  |  |  | |  | | 12 | -37 | | -44 | | 3.81 | 18 |
|  | l |  | |  |  |  | |  | | -6 | -37 | | -41 | | 4.01 | 27 |
| Cerebellum | l | -15 | | -88 | -35 | 4.77 | | 150 | | -21 | -82 | | -41 | | 4.30 | 125 |
|  | l | -15 | | -79 | -44 | 3.73 | | 13 | |  |  | |  | |  |  |
|  | l | -9 | | -91 | -29 | 3.72 | | 18 | |  |  | |  | |  |  |
|  | r |  | |  |  |  | |  | | 15 | -79 | | -44 | | 4.20 | 66 |
| OFC/ Frontal Pole | r |  | |  |  |  | |  | | 24 | 32 | | -17 | | 3.80 | 9 |
|  | l |  | |  |  |  | |  | | -39 | 29 | | -14 | | 3.55 | 14 |
| ITG | l |  | |  |  |  | |  | | -48 | -64 | | -23 | | 3.72 | 20 |
|  | Controls > ASD | | | | | | | | | | | | | | | |
|  |  | | x | | | | y | | z | Z | | cluster  size | |  | | |
|  | - | | | | | | | | | | | | | | | |
|  | ASD > Controls | | | | | | | | | | | | | | | |
|  | - | | | | | | | | | | | | | | | |
|  | | | | | | | | | | | | | | | | |
|  | | Non-vocal sounds > vocal sounds | | | | | | | | | | | | | | |
|  | | Controls | | | | | | | | ASD | | | | | | |
| Region | | x | | Y | z | *Z* | | cluster  size | | x | y | | z | | *Z* | cluster  size |
| LOC | r | 21 | | -85 | 34 | 4.17 | | 95 | |  |  | |  | |  |  |
|  |  | -39 | | -76 | 22 | 4.36 | | 51 | |  |  | |  | |  |  |
| Fusiform Cortex | l | -30 | | -34 | -26 | 4.48 | | 106 | | -27 | -40 | | -20 | | 4.66 | 69 |
|  | r |  | |  |  |  | |  | | 27 | -37 | | -17 | | 3.58 | 12 |
| Parahippocampus | r | 24 | | -28 | -17 | 4.28 | | 28 | |  |  | |  | |  |  |
| Frontal Pole | r | 27 | | 50 | 31 | 4.21 | | 54 | |  |  | |  | |  |  |
|  |  | 36 | | 41 | 19 | 3.58 | | 17 | |  |  | |  | |  |  |
| Cingulate Gyrus | l | -9 | | -52 | 4 | 3.53 | | 9 | |  |  | |  | |  |  |
| Precuneous | l | -3 | | -40 | 52 | 3.78 | | 42 | |  |  | |  | |  |  |
| SMG |  | 54 | | -37 | 37 | 3.86 | | 35 | |  |  | |  | |  |  |
|  |  |  | |  |  |  | |  | |  |  | |  | |  |  |
|  | Controls > ASD  (≙ ASD > Controls for vocal sounds > non-vocal sounds for) | | | | | | | | | | | | | | | |
|  |  | | x | | | | y | | z | Z | | cluster  size | |  | | |
| Central Operculum | r | | 45 | | | | 2 | | 7 | 3.98 | | 13 | |  | | |
| STS/STG | l | | -60 | | | | -31 | | 10 | 3.90 | | 29 ^b^ | |  | | |
| Precuneous/ Postcentral Gyrus | r | | 9 | | | | -40 | | 55 | 3.75 | | 19 | |  | | |

| **Voice identity recognition experiment** | | | | | | | | | | | | | | | |
| --- | --- | --- | --- | --- | --- | --- | --- | --- | --- | --- | --- | --- | --- | --- | --- |
|  | | Voice identity task | | | | | | | | | | | | | |
|  | | Controls | | | | | | | ASD | | | | | | |
| Region | | x | y | z | *Z* | | cluster  size | | x | y | | z | | *Z* | cluster  size |
| STS/STG | l | -63 | -19 | 4 | 6.06 | | 1076 ^b^ | | -57 | -22 | | 7 | | 6.58 | 1000 ^b^ |
|  | r | 54 | -16 | 1 | 5.73 | | 900 ^b^ | | 66 | -25 | | 10 | | 6.42 | 893 ^b^ |
| Cerebellum | l | -12 | -79 | -32 | 5.46 | | 436 | | -15 | -76 | | -44 | | 4.09 | 22 |
|  | r | 27 | -58 | -29 | 4.72 | | 178 | | 27 | -58 | | -26 | | 4.49 | 71 |
|  |  | 24 | -64 | -44 | 3.94 | | 74 | |  |  | |  | |  |  |
| Insula | l | -30 | 26 | 7 | 5.12 | | 837 | | -27 | 23 | | 4 | | 4.09 | 99 |
|  | r |  |  |  |  | |  | | 30 | 23 | | 4 | | 5.12 | 167 |
| SMA | l | -9 | -1 | 58 | 5.07 | | 348 | | -6 | 5 | | 58 | | 5.19 | 333 |
| MFG/ Frontal Pole | r | 42 | 32 | 28 | 5.06 | | 849 | |  |  | |  | |  |  |
| Frontal Pole | l | -48 | 41 | -2 | 3.20 | | 11 | |  |  | |  | |  |  |
| Precentral Gyrus | l | -51 | -4 | 49 | 4.81 | | 23 | | -51 | -7 | | 43 | | 4.38 | 115 |
|  |  |  |  |  |  | |  | | 54 | 2 | | 43 | | 4.63 | 255 |
| Angular Gyrus | r | 39 | -52 | 40 | 4.12 | | 41 | |  |  | |  | |  |  |
| Putamen | l |  |  |  |  | |  | | -21 | -1 | | 10 | | 4.11 | 58 |
| Postcentral Gyrus | l |  |  |  |  | |  | | -51 | -31 | | 55 | | 3.85 | 63 |
| Thalamus | r | 12 | -10 | 10 | 3.80 | | 77 | |  |  | |  | |  |  |
| IFG | l |  |  |  |  | |  | | -48 | 20 | | 25 | | 3.66 | 26 |
| Brain-Stem | r | 9 | -19 | -11 | 3.58 | | 12 | |  |  | |  | |  |  |
| Angular Gyrus | l | -36 | -52 | 43 | 3.48 | | 17 | |  |  | |  | |  |  |
|  | r |  |  |  |  | |  | | 39 | -52 | | 43 | | 3.76 | 25 |
|  | Controls > ASD | | | | | | | | | | | | | | |
|  |  | x | | | | y | | z | Z | | cluster  size | |  | | |
| Precuneous | r | 3 | | | | -61 | | 28 | 4.16 | | 123 | |  | | |
| Cerebellum | l | -24 | | | | -76 | | -32 | 3.92 | | 131 | |  | | |
|  | r | 27 | | | | -85 | | -41 | 3.53 | | 46 | |  | | |
| MTG | r | 54 | | | | -31 | | -11 | 3.89 | | 14 | |  | | |
| Frontal Pole | l | -6 | | | | 65 | | 16 | 3.75 | | 14 | |  | | |
|  | ASD > Controls | | | | | | | | | | | | | | |
|  | - | | | | | | | | | | | | | | |
|  | | | | | | | | | | | | | | | |
|  | | Voice identity task > speech task | | | | | | | | | | | | | |
|  | | Controls | | | | | | | ASD | | | | | | |
| Region | | x | y | z | *Z* | | cluster  size | | x | y | | z | | *Z* | cluster  size |
| Precuneous | r | 9 | -67 | 46 | 609 | | 849 | |  |  | |  | |  |  |
| MFG | r | 30 | 14 | 49 | 5.85 | | 2294 | | 42 | 32 | | 31 | | 4.74 | 116 |
|  | l | -51 | 26 | 31 | 4.44 | | 131 | |  |  | |  | |  |  |
|  |  | -27 | 8 | 52 | 3.97 | | 29 | |  |  | |  | |  |  |
| Cerebellum | l | -39 | -67 | -29 | 5.21 | | 483 | |  |  | |  | |  |  |
|  |  | -9 | -55 | -44 | 4.17 | | 14 | |  |  | |  | |  |  |
| Insula | l | -33 | 17 | -5 | 4.53 | | 47 | |  |  | |  | |  |  |
| Frontal Pole | l | -30 | 47 | 13 | 4.48 | | 138 | |  |  | |  | |  |  |
| Angular Gyrus | l | -39 | -55 | 43 | 4.14 | | 25 | |  |  | |  | |  |  |
|  | r | 54 | -46 | 19 | 3.72 | | 11 | | 39 | -52 | | 43 | | 3.91 | 44 |
|  | Controls > ASD  (≙ ASD > Controls for speech task > voice identity task) | | | | | | | | | | | | | | |
|  |  | x | | | | y | | z | Z | | cluster  size | |  | | |
| Precuneous | r | 3 | | | | -73 | | 43 | 4.65 | | 266 | |  | | |
| Cerebellum | r | 18 | | | | -52 | | -41 | 4.29 | | 25 | |  | | |
|  | l | -6 | | | | -55 | | -41 | 3.78 | | 34 | |  | | |
|  | l | -39 | | | | -64 | | -26 | 3.67 | | 16 | |  | | |
| STS/STG | r | 51 | | | | -19 | | -2 | 3.63 | | 14 | |  | | |
|  | r | 66 | | | | -34 | | 7 | 3.40 | | 9 | |  | | |
| Cingulate Gyrus | r | 6 | | | | 29 | | 37 | 3.58 | | 9 | |  | | |
| Occipital Pole | r | 6 | | | | -94 | | 4 | 3.55 | | 56 | |  | | |
| MFG | r | 30 | | | | 14 | | 46 | 3.52 | | 19 | |  | | |
| MFG/SFG | l | -24 | | | | 11 | | 52 | 3.43 | | 14 | |  | | |
|  |  |  | | | |  | |  |  | |  | |  | | |

|  | | Correlation voice identity with task performance | | | | | | | | | | | | | |
| --- | --- | --- | --- | --- | --- | --- | --- | --- | --- | --- | --- | --- | --- | --- | --- |
|  | | Controls | | | | | | | ASD | | | | | | |
| Region | | x | y | z | *Z* | | cluster  size | | x | y | | z | | *Z* | cluster  size |
| STS/STG | r | 45 | -34 | 7 | 3.75 | | 67 ^c^ | | - | | | | | | |
| Precuneous | r | 6 | -58 | 37 | 3.39 | | 12 | |  |  | |  | |  |  |
| MTG | r | 48 | -28 | -5 | 3.35 | | 14 | |  |  | |  | |  |  |
|  | Controls > ASD | | | | | | | | | | | | | | |
|  |  | x | | | | y | | z | Z | | cluster  size | |  | | |
|  | - | | | | | | | | | | | | | | |
|  | ASD > Controls | | | | | | | | | | | | | | |
|  | - | | | | | | | | | | | | | | |

|  | | Speech task | | | | | | | | | | | | | |
| --- | --- | --- | --- | --- | --- | --- | --- | --- | --- | --- | --- | --- | --- | --- | --- |
|  | | Controls | | | | | | | ASD | | | | | | |
| Region | | x | y | z | *Z* | | cluster  size | | x | y | | z | | *Z* | cluster  size |
| SMA | l | -9 | -1 | 58 | 6.18 | | 275 | |  |  | |  | |  |  |
| STS/STG | l | -63 | -19 | 4 | 6.00 | | 2364 ^b^ | | -57 | -22 | | 7 | | 6.64 | 2612 ^b^ |
|  | r | 54 | -16 | 4 | 5.56 | | 883 ^a,b^ | | 66 | -25 | | 10 | | 6.32 | 984 ^b^ |
| Cerebellum | r | 21 | -58 | -23 | 4.77 | | 332 | | 24 | -61 | | -23 | | 5.41 | 180 |
|  | r |  |  |  |  | |  | | 18 | -64 | | -44 | | 4.60 | 25 |
|  | l | -12 | -79 | -32 | 4.23 | | 47 | | -15 | -76 | | -44 | | 4.17 | 23 |
|  | l | -30 | -58 | -29 | 3.47 | | 21 | | -30 | -58 | | -29 | | 3.91 | 37 |
| IFG | r | 39 | 23 | 13 | 4.35 | | 240 | |  |  | |  | |  |  |
| SMG | l | -48 | -37 | 40 | 3.96 | | 56 | |  |  | |  | |  |  |
| Precentral Gyrus | r |  |  |  |  | |  | | 54 | 2 | | 43 | | 4.87 | 65 |
|  | l | -24 | -7 | 52 | 3.94 | | 29 | |  |  | |  | |  |  |
| MFG | r | 42 | 26 | 28 | 3.77 | | 42 | |  |  | |  | |  |  |
| Insula/ OFC | r |  |  |  |  | |  | | 33 | 23 | | 7 | | 4.78 | 101 |
|  | l |  |  |  |  | |  | | -27 | 23 | | 1 | | 4.15 | 95 |
| Putamen | r |  |  |  |  | |  | | 21 | 2 | | 10 | | 4.37 | 144 |
|  |  |  |  |  |  | |  | |  |  | |  | |  |  |
|  | Controls > ASD | | | | | | | | | | | | | | |
|  |  | x | | | | y | | z | Z | | cluster  size | |  | | |
| Cerebellum | r | 33 | | | | -76 | | -41 | 3.66 | | 24 | |  | | |
|  | ASD > Controls | | | | | | | | | | | | | | |
|  | - | | | | | | | | | | | | | | |

|  | | Speech task > voice identity task | | | | | | | | | | | | | |
| --- | --- | --- | --- | --- | --- | --- | --- | --- | --- | --- | --- | --- | --- | --- | --- |
|  | | Controls | | | | | | | ASD | | | | | | |
| Region | | x | y | z | *Z* | | cluster  size | | x | y | | z | | *Z* | cluster  size |
| ITG/MTG | l | -48 | -49 | -5 | 5.09 | | 494 | | -45 | -61 | | -8 | | 4.63 | 392 |
| LOC | r | 54 | -61 | -2 | 3.62 | | 26 | |  |  | |  | |  |  |
| Pallidum | l | -24 | -4 | -5 | 4.95 | | 921 | |  |  | |  | |  |  |
|  | r |  |  |  |  | |  | | 24 | -1 | | -5 | | 3.85 | 29 |
| Cerebellum | r | 18 | -61 | -20 | 4.22 | | 91 | | 18 | -52 | | -41 | | 4.04 | 67 |
|  |  |  |  |  |  | |  | | 33 | -46 | | -29 | | 3.34 | 10 |
|  | l |  |  |  |  | |  | | -18 | -58 | | -23 | | 3.84 | 21 |
| Hippocampus | r | 33 | -22 | -14 | 4.21 | | 30 | |  |  | |  | |  |  |
| Amygdala | r | 27 | -1 | -14 | 4.16 | | 63 | |  |  | |  | |  |  |
| Occipital Pole | l |  |  |  |  | |  | | -9 | -91 | | -5 | | 4.06 | 50 |
| Precentral Gyrus | r | 63 | 5 | 19 | 4.07 | | 16 | | 60 | 2 | | 28 | | 4.14 | 34 |
|  | l | -33 | -16 | 67 | 3.58 | | 41 | | -51 | -4 | | 34 | | 4.12 | 109 |
| Brain-Stem | l |  |  |  |  | |  | | -12 | -22 | | 46 | | 3.49 | 22 |
| Thalamus | l |  |  |  |  | |  | | -9 | -19 | | 4 | | 3.55 | 22 |
| STS/STG | r |  |  |  |  | |  | | 51 | -19 | | 10 | | 3.95 | 81 ^a^ |
|  | l | -51 | -7 | -14 | 3.54 | | 20 | |  |  | |  | |  |  |
| Postcentral Gyrus | r | 42 | -19 | 37 | 3.48 | | 15 | |  |  | |  | |  |  |
| Cingulate Gyrus | r | 12 | -7 | 40 | 3.74 | | 11 | | 6 | -34 | | 40 | | 3.57 | 36 |
|  |  |  |  |  |  | |  | | 6 | -1 | | 37 | | 3.30 | 23 |
|  | l | -6 | 8 | 37 | 3.40 | | 9 | |  |  | |  | |  |  |
| ITG | l | -48 | -7 | -29 | 3.66 | | 18 | |  |  | |  | |  |  |
| SMA | l | -3 | -13 | 55 | 3.63 | | 29 | |  |  | |  | |  |  |
| Frontal Pole | l |  |  |  |  | |  | | -6 | 59 | | 22 | | 3.50 | 17 |
| Lingual Gyrus | r | 21 | -61 | -5 | 3.55 | | 16 | | 3 | -61 | | 1 | | 3.65 | 41 |
|  |  |  |  |  |  | |  | | 12 | -82 | | -5 | | 3.59 | 55 |
| Parietal Operculum | r | 51 | -25 | 22 | 3.47 | | 18 | |  |  | |  | |  |  |
| Central Operculum | r |  |  |  |  | |  | | 36 | 8 | | 10 | | 3.73 | 15 |
| Precuneous/ Cingulate Gyrus | l | -6 | -52 | 13 | 3.47 | | 18 | |  |  | |  | |  |  |
| Precuneous | r |  |  |  |  | |  | | 30 | -58 | | 13 | | 4.06 | 59 |
|  | Controls > ASD  (≙ ASD > Controls for voice identity task > speech task) | | | | | | | | | | | | | | |
|  |  | x | | | | y | | z | Z | | cluster  size | |  | | |
|  | - | | | | | | | | | | | | | | |

Coordinates represent local activation maxima in MNI space (in mm) for the whole brain. Cluster size represents the number of voxels within a cluster. Clusters are reported that reached *p* = 0.001 uncorrected (peak-level) and a cluster size of more than 8 voxels. Regions were labelled based on a standard anatomical atlas (Harvard-Oxford cortical and subcortical structural atlases; Desikan *et al.*, 2006) implemented in FSL (Smith *et al.*, 2004; http://www.fmrib.ox.ac.uk/fsl/fslview). If not labelled in FSL or when the labelling was ambiguous, we additionally used atlases implemented in the WFU Pick Atlas tool (Maldjian et al., 2003). r = right; l = left; STS/STG = Superior Temporal Sulcus/Superior Temporal Gyrus; SMA = Supplementary Motor Area; LOC = Lateral Occipital Cortex; SMG = Supramarginal Gyrus; MFG = Middle Frontal Gyrus; MTG = Middle Temporal Gyrus; IFG = Inferior Frontal Gyrus; ITG = Inferior Temporal Gyrus; OFC = Orbital Frontal Cortex; SFG = Superior Frontal Gyrus; ^a^ peak coordinate in Heschl’s Gyrus; ^b^ peak coordinate in Planum Temporale; ^c^ peak coordinate in SMG.

Supplementary Table 4

Coordinates for significant BOLD-responses in the voice identity recognition experiment for ROI analyses with the right precuneus, the left middle temporal gyrus and the right medial temporal pole (*p* < 0.05 FWE- corrected at peak level for the region of interest, Bonferroni corrected for three ROIs).

|  | **Voice identity task** | | | | | | | | | | |
| --- | --- | --- | --- | --- | --- | --- | --- | --- | --- | --- | --- |
|  | Controls | | | | |  | ASD | | | | |
| supramodal regions | x | y | z | *Z* | cluster  size |  | x | y | z | *Z* | cluster  size |
| r Precuneous |  |  | - |  |  |  |  |  | - |  |  |
| l Middle Temporal Gyrus |  |  | - |  |  |  |  |  | - |  |  |
| r Medial Temporal Pole |  |  | - |  |  |  |  |  | - |  |  |
|  | Controls > ASD | | | | |  | ASD > Controls | | | | |
| r Precuneous | 3 | -61 | 28 | 4.16 | 118 |  |  |  | - |  |  |
| l Middle Temporal Gyrus |  |  | - |  |  |  |  |  | - |  |  |
| r Medial Temporal Pole |  |  | - |  |  |  |  |  | - |  |  |

|  | **Voice identity task > speech task** | | | | | | | | | | |
| --- | --- | --- | --- | --- | --- | --- | --- | --- | --- | --- | --- |
|  | Controls | | | | |  | ASD | | | | |
| supramodal regions | x | y | z | *Z* | cluster  size |  | x | y | z | *Z* | cluster  size |
| r Precuneous | 6 | -61 | 34 | 4.97 | 118 |  |  |  | - |  |  |
| l Middle Temporal Gyrus |  |  | - |  |  |  |  |  | - |  |  |
| r Medial Temporal Pole |  |  | - |  |  |  |  |  | - |  |  |
|  | Controls > ASD | | | | |  | ASD > Controls | | | | |
| r Precuneous | 6 | -61 | 34 | 3.71 | 118 |  |  |  | - |  |  |
| l Middle Temporal Gyrus |  |  | - |  |  |  |  |  | - |  |  |
| r Medial Temporal Pole |  |  | - |  |  |  |  |  | - |  |  |

|  | **Correlation voice identity with task performance** | | | | | | | | | | |
| --- | --- | --- | --- | --- | --- | --- | --- | --- | --- | --- | --- |
|  | Controls | | | | |  | ASD | | | | |
| supramodal regions | x | y | z | *Z* | cluster  size |  | x | y | z | *Z* | cluster  size |
| r Precuneous | 3 | -58 | 34 | 3.30 | 118 |  |  |  | - |  |  |
| l Middle Temporal Gyrus |  |  | - |  |  |  |  |  | - |  |  |
| r Medial Temporal Pole |  |  | - |  |  |  |  |  | - |  |  |
|  | Controls > ASD | | | | |  | ASD > Controls | | | | |
| r Precuneous |  |  | - |  |  |  |  |  | - |  |  |
| l Middle Temporal Gyrus |  |  | - |  |  |  |  |  | - |  |  |
| r Medial Temporal Pole |  |  | - |  |  |  |  |  | - |  |  |

Coordinates represent local activation maxima in MNI space (in mm). Cluster size represents the number of voxels within a cluster. r = right; l = left.

Supplementary Table 5

Group comparisons of the average movement (in mm) for all directions (x, y, and z) for the voice identity recognition and the vocal sound experiment.

|  | | ASD | |  | Controls | |  |
| --- | --- | --- | --- | --- | --- | --- | --- |
| Direction | Average movement (mm) | *M* | *SE* |  | *M* | *SE* | *p* |
|  |  |  |  |  |  |  |  |
| Voice identity recognition experiment (507 images) | | | | | | | |
|  |  |  |  |  |  |  |  |
| xyz | Directed | 0.14 | 0.11 |  | 0.16 | 0.06 | 0.853 |
|  | Absolute value | 0.32 | 0.08 |  | 0.24 | 0.04 | 0.388 |
|  |  |  |  |  |  |  |  |
| x | Directed | 0.10 | 0.11 |  | -0.18 | 0.11 | 0.433 |
|  | Absolute value | 0.35 | 0.07 |  | 0.33 | 0.06 | 0.829 |
|  |  |  |  |  |  |  |  |
| y | Directed | 0.24 | 0.06 |  | 0.22 | 0.04 | 0.749 |
|  | Absolute value | 0.29 | 0.03 |  | 0.24 | 0.03 | 0.246 |
|  |  |  |  |  |  |  |  |
| z | Directed | 0.08 | 0.26 |  | 0.30 | 0.16 | 0.485 |
|  | Absolute value | 0.74 | 0.18 |  | 0.49 | 0.12 | 0.255 |
|  |  |  |  |  |  |  |  |
|  | |  |  |  |  |  |  |
| Vocal sound experiment (60 images) | | | | | | | |
|  |  |  |  |  |  |  |  |
| xyz | Directed | 0.05 | 0.04 |  | -0.07 | 0.05 | 0.105 |
|  | Absolute value | 0.14 | 0.02 |  | 0.17 | 0.04 | 0.460 |
|  |  |  |  |  |  |  |  |
| x | Directed | -0.06 | 0.08 |  | -0.17 | 0.08 | 0.374 |
|  | Absolute value | 0.22 | 0.05 |  | 0.30 | 0.05 | 0.339 |
|  |  |  |  |  |  |  |  |
| y | Directed | 0.02 | 0.03 |  | 0.01 | 0.06 | 0.931 |
|  | Absolute value | 0.10 | 0.02 |  | 0.13 | 0.04 | 0.558 |
|  |  |  |  |  |  |  |  |
| z | Directed | 0.19 | 0.09 |  | -0.05 | 0.16 | 0.214 |
|  | Absolute value | 0.30 | 0.06 |  | 0.45 | 0.11 | 0.246 |
|  |  |  |  |  |  |  |  |

Directions: x = medial-lateral axis; y = anterior-posterior axis; z = dorsal-ventral axis. Directed values represent the average of movements along positive and negative direction of each axis. Absolute values represent the absolute movement along one axis independent of the direction (positive or negative) of the movement along the axis. We compared the individual average head movement (in mm) over all images acquired in each experiment for each direction (x, y, and z) and over all directions (average from movements in the x, y, and z- direction). Independent *t*-tests revealed that there were no differences in the amount of movement between the groups for any of the directions for any of the experiments. *M* = mean; *SE* = standard error.

**Supplementary References**

Belin, P., Zatorre, R. J., Lafaille, P., Ahad, P. & Pike, B. (2000). Voice-selective areas in human auditory cortex. *Nature*, **403**(6767), 309-312.

Blank, H., Anwander, A. & von Kriegstein, K. (2011). Direct structural connections between voice- and face-recognition areas. *Journal of Neuroscience*, **31**(36), 12906-12915.

Blank, H., Wieland, N. & von Kriegstein, K. (2014). Person recognition and the brain: merging evidence from patients and healthy individuals. *Neuroscience and Biobehavioral Reviews*, **47C**, 717-734.

Bölte, S. & Poustka, F. (2006). Fragebogen zur Sozialen Kommunikation (FSK). Bern: Verlag Hans Huber.

Bölte, S., Rühl, D., Schmötzer, G. & Poustka, F. (2003). Diagnostisches Interview für Autiamus – Revidiert (ADI-R). Bern: Verlag Hans Huber.

Brickenkamp, R. (2002). Test d2 - Aufmerksamkeits-Belastung-Test (d2). Göttingen: Hogrefe.

Desikan, R. S., Segonne, F., Fischl, B., Quinn, B. T., Dickerson, B. C., Blacker, D., Buckner, R. L., Dale, A. M., Maguire, R. P., Hyman, B. T., Albert, M. S. & Killiany, R. J. (2006). An automated labeling system for subdividing the human cerebral cortex on MRI scans into gyral based regions of interest. *Neuroimage*, **31**(3), 968-980.

Friston, K., Ashburner, A., Kiebel, S., Nichols, T. & Penny W. (2007). Statistical Parametric Mapping: the analysis of functional brain images. London: Academic Press.

Jezzard, P. & Balaban, R. S. (1995). Correction for geometric distortions in echoplanar images from B0 field variations. *Magnetic Resonance Imaging*, **34**(1), 65-73.

Maldjian, J. A., Laurienti, P. J., Kraft, R. A., & Burdette, J. H. (2003). An automated method for neuroanatomic and cytoarchitectonic atlas-based interrogation of fMRI data sets. *Neuroimage, 19*(3), 1233-1239.

Mugler, J. P. & Brookeman, J. R. (1990). Three-dimensional magnetization-prepared rapid gradient-echo imaging (3D MP RAGE). *Magnetic Resonance Medicine*, **15**(1), 152-157.

Rutter, M., Bailey, A. & Lord, C. (2003). Social Communication Questionnaire (SCQ). Los Angeles, CA: Western Psychological Services.

Rühl, D., Bölte, S., Feineis-Matthews, S. & Poustka, F. (2004). Diagnostische Beobachtungsskala für Autistische Störungen (ADOS). Bern: Verlag Hans Huber.

Smith, S. M., Jenkinson, M., Woolrich, M. W., Beckmann, C. F., Behrens, T. E., Johansen-Berg, H. *et al.* (2004). Advances in functional and structural MR image analysis and implementation as FSL. *Neuroimage*, **23**(Suppl 1), S208-219.

von Aster, M., Neubauer, A. & Horn, R. (2006). Wechsler Intelligenztest für Erwachsene (WIE). Frankfurt/M: Harcourt Test Services.

von Kriegstein, K., Eger, E., Kleinschmidt, A. & Giraud, A. L. (2003). Modulation of neural responses to speech by directing attention to voices or verbal content. *Cognitive Brain Research*, **17**(1), 48-55.

von Kriegstein, K. & Giraud, A. L. (2004). Distinct functional substrates along the right superior temporal sulcus for the processing of voices. *Neuroimage*, **22**(2), 948-955.

von Kriegstein, K., Kleinschmidt, A., Sterzer, P. & Giraud, A. L. (2005). Interaction of face and voice areas during speaker recognition. *Journal of Cognitive Neuroscience*, **17**(3), 367-376.

Wechsler, D. (1997). Wechsler Adult Intelligence Scale (WAIS-III). San Antonio, TX: The Psychological Corporation.
